# Natto consumption suppresses atherosclerotic plaque progression in LDL receptor-deficient mice transplanted with iRFP-expressing hematopoietic cells

**DOI:** 10.1038/s41598-023-48562-y

**Published:** 2023-12-18

**Authors:** Takeshi Kawamata, Arata Wakimoto, Takanobu Nishikawa, Masaya Ikezawa, Michito Hamada, Yuri Inoue, Kaushalya Kulathunga, Filiani Natalia Salim, Maho Kanai, Teppei Nishino, Kyle Gentleman, Chang Liu, Bryan J. Mathis, Nozomu Obana, Shinji Fukuda, Satoru Takahashi, Yuki Taya, Satoshi Sakai, Yuji Hiramatsu

**Affiliations:** 1 Tsukuba Medical Center Hospital, 1-3-1, Amakubo, Tsukuba, Ibaraki 305-8558 Japan; 2 Doctoral Program in Medical Sciences, Graduate School of Comprehensive Human Sciences, University of Tsukuba, 1-1-1, Tennodai, Tsukuba, Ibaraki 305-8575 Japan; 3 Department of Anatomy and Embryology, Faculty of Medicine, University of Tsukuba, 1-1-1, Tennodai, Tsukuba, Ibaraki 305-8575 Japan; 4 Ph.D. Program in Human Biology, School of Integrative and Global Majors, University of Tsukuba, 1-1-1, Tennodai, Tsukuba, Ibaraki 305–8575 Japan; 5 Department of Natto Research and Development, Takanofoods Corporation, 1542, Noda, Omitama, Ibaraki 311-3411 Japan; 6 Laboratory Animal Resource Center, Faculty of Medicine, University of Tsukuba, 1-1-1, Tennodai, Tsukuba, Ibaraki 305-8575 Japan; 7 Integrated Master of Science Natural Sciences, University of Southampton, Highfield, Southampton, SO17 1BJ Hampshire UK; 8 Department of Cardiovascular Surgery, Faculty of Medicine, University of Tsukuba, 1-1-1, Tennodai, Tsukuba, Ibaraki 305-8575 Japan; 9 Department of Cardiovascular Medicine, Faculty of Medicine, University of Tsukuba, 1-1-1, Tennodai, Tsukuba, Ibaraki 305-8575 Japan; 10 Transborder Medical Research Center, Faculty of Medicine, University of Tsukuba, Tsukuba, Ibaraki Japan; 11 Microbiology Research Center for Sustainability, University of Tsukuba, Tsukuba, Ibaraki Japan; 12 Institute for Advanced Biosciences, Keio University, 246-2 Mizukami, Kakuganji, Tsuruoka-shi, Yamagata 997-0052 Japan; 13 Gut Environmental Design Group, Kanagawa Institute of Industrial Science and Technology, 3-25-13 Tonomachi, Kawasaki-ku, Kawasaki, Kanagawa 210-0821 Japan; 14 Transborder Medical Research Center, University of Tsukuba, 1-1-1 Tennodai, Tsukuba-shi, Ibaraki 305-8575 Japan; 15 Department of Physiology, Faculty of Medicine, Sabaragamuwa University of Sri Lanka, P.O. Box 01, Hidellana, Ratnapura, Sri Lanka; 16 Centre for Medical Science and Technology and Healthcare Equity, Parahyangan Catholic University, Bandung, 40141 Indonesia; 17 Magister Program of Biomedical Sciences, Universitas Padjadjaran, Sumedang, 45363 Indonesia; 18 Laboratory for Regenerative Microbiology, Juntendo University Graduate School of Medicine, 2-1-1 Hongo, Bunkyo-ku, Tokyo 113-8421 Japan; 19 Faculty of Health Sciences, Tsukuba University of Technology, 4-12-7, Kasuga, Tsukuba, Ibaraki 305-8521 Japan

**Keywords:** Atherosclerosis, Animal disease models

## Abstract

Natto, known for its high vitamin K content, has been demonstrated to suppress atherosclerosis in large-scale clinical trials through a yet-unknown mechanism. In this study, we used a previously reported mouse model, transplanting the bone marrow of mice expressing infra-red fluorescent protein (iRFP) into LDLR-deficient mice, allowing unique and non-invasive observation of foam cells expressing iRFP in atherosclerotic lesions. Using 3 natto strains, we meticulously examined the effects of varying vitamin K levels on atherosclerosis in these mice. Notably, high vitamin K natto significantly reduced aortic staining and iRFP fluorescence, indicative of decreased atherosclerosis. Furthermore, mice administered natto showed changes in gut microbiota, including an increase in natto bacteria within the cecum, and a significant reduction in serum CCL2 expression. In experiments with LPS-stimulated macrophages, adding natto decreased CCL2 expression and increased anti-inflammatory cytokine IL-10 expression. This suggests that natto inhibits atherosclerosis through suppression of intestinal inflammation and reduced CCL2 expression in macrophages.

## Introduction

Atherosclerosis is a chronic, multifactorial disease characterized by the accumulation of lipids, inflammatory cells, and extracellular matrix components within the arterial wall^1^. The resulting atherosclerotic plaques can progress over time, eventually leading to cardiovascular events such as myocardial infarction and stroke^2^. Despite advances in atherosclerosis prevention and treatment, it remains the leading cause of death worldwide^3^ that also induces dysfunction within the cardiovascular system, jeopardizes overall health, significantly diminishes the quality of life, and increases the cost of healthcare, making it a crucial, global public health issue.

A key aspect of atherosclerosis research involves the localization of macrophages in atherogenic lesions, making them valuable markers for in vivo imaging^4,5^. Exploiting this phenomenon was detailed in our recent publication, which highlighted the utility of near-infrared fluorescent protein (iRFP) to identify potential drugs or foods capable of reducing atherosclerotic lesions. This non-invasive imaging approach does not require any injections in mice, making it an attractive tool for evaluating therapeutic interventions^5,6^.

Natto, a traditional Japanese food made from fermented soybeans, is a rich source of vitamin K2 and has been repeatedly shown to benefit the cardiovascular system^7^. Epidemiological studies have suggested a possible inverse association between vitamin K2 intake and cardiovascular disease risk^8^ by specifically inhibiting arterial calcification, enhancing arterial elasticity, and modulating inflammation^9^. However, the underlying mechanisms remain unclear and additional preclinical and clinical studies are needed to evaluate the potential benefits of vitamin K2 against atherosclerosis. However, patients on anticoagulation therapy are given warfarin, which inhibits vitamin K, thereby necessitating a limited intake of this vitamin. This creates a dilemma, as natto, a food beneficial against atherosclerosis and rich in vitamin K2, should otherwise be an ideal dietary choice.

Natto additionally benefits the gut microbiota, an emergent factor in the development and progression of atherosclerosis^10^. Recent studies have shown that changes in gut microbiota composition and diversity can impact immune response, inflammation, and lipid metabolism, all of which are implicated in atherosclerosis^11^. Furthermore, gut microbiota-derived metabolites, such as trimethylamine N-oxide (TMAO), have been shown as drivers of atherosclerotic pathogenesis^12^. Additionally, chemokines, such as CCL2/MCP1 (a key regulator of macrophage recruitment in atherosclerotic plaques), are modulated by gut microbiota composition and metabolites^10^. Thus, understanding the complex interplay between gut microbiota and atherosclerosis may lead to new therapeutic strategies.

The current study evaluated the impact of natto consumption on atherosclerotic progression using an in vivo murine imaging model and strains of natto with varying vitamin K2 levels developed originally to accommodate patients on anticoagulation therapy. These strains facilitated the creation of three types of natto: high vitamin K natto (HVK), normal natto (NN), and low vitamin K natto (LVK). The influence of each type on atherosclerosis was then assessed using iRFP in a non-invasive imaging method^6^.

Findings suggest that natto intake therapeutically affects atherosclerosis by modulating gut microbiota composition and regulating the expression of pro-atherosclerotic cytokines and chemokines, such as CCL2. Macrophage gene expression analysis indicated that vitamin K2, surfactin, and the bacteria themselves play key roles in these effects. Notably, employing iRFP-expressing hematopoietic cells enabled direct visualization of natto's heightened efficacy in treating atherosclerosis.

## Results

### Quantitative analysis of natto variants

Initially, three distinct natto variants were developed, distinguished by their vitamin K2 concentrations. These variants were created using different strains of bacteria involved natto fermentation and were comprised of high vitamin K2 natto (HVK), normal natto (NN), and low vitamin K2 natto (LVK). Each was tested against high-cholesterol diet (HCD) controls for impact on atherosclerotic pathogenesis with regard to modulation of macrophage activation and lesion size.

Our results showed no significant differences among the three natto types in terms of water, protein, fat, fiber, ash, and soluble non-nitrogenous substances. However, striking disparities were apparent in the vitamin K2 content: HVK contained the highest quantity with 199 μg/100 g, followed by NN with 93 μg/100 g, and LVK with 30 μg/100 g. No vitamin K2 was detected in the HCD (Supplementary Fig. 1A). Furthermore, nattokinase activity was highest in HVK at 110 FU/g, compared to NN at 82 FU/g, and LVK at 38 FU/g.

Additionally, the bacterial count was greatest in HVK natto at 19,500 (×10^6^ cfu/g), followed by NN natto at 13,700 (×10^6^ cfu/g) and LVK natto at 638 (×10^6^ cfu/g). In terms of polyglutamic acid (PGA) content, HVK also showed the highest quantity at 12.2 mg/g, with NN at 9.3 mg/g and LVK at 4.9 mg/g (Supplementary Fig. 1B).

Our findings showed that HVK natto contained the highest amount of vitamin K2, exhibited the greatest nattokinase activity, had the largest bacteria count, and had the highest PGA content. These parameters, except for PGA, have previously been shown to influence atherosclerosis and inflammation suppression^13^. Conversely, LVK natto was found to have the lowest values for each surveyed attribute. To ascertain the influence these parameters could have on atherosclerotic development, each natto type was tested against a control diet in a murine atherosclerosis model. Between these analyses, we compared the dietary intake and body weight measurements of the different feeding groups (HCD, HCD + HVK, HCD + LVK, HCD + NN), taken weekly (Supplementary Fig. 2A, B). The results did not reveal any statistically significant differences among the groups, which suggests that neither natto in the diet nor the differences in vitamin K2 content had a discernible impact on body weight or food consumption.

### Effect of natto types on atherosclerotic formation in a murine model

Next, we utilized bone marrow-transplanted mice expressing iRFP (*iRFP → LDLR*^*−*/−^) generated using a previously described method for imaging atherosclerosis^6^. In these mice, iRFP is expressed in blood cells, and the fluorescence of atherosclerotic lesions has been previously reported to precisely indicate the size of lesions^6^. We induced atherosclerosis in these mice by a high-cholesterol diet (HCD) for nine weeks, during which we performed weekly in vivo imaging to observe iRFP signal. At the end of the ninth week, we harvested aortas after sacrifice and performed Oil red O staining to visualize arterial lipid accumulation (Supplementary Fig. 2C).

**Figure 1:**
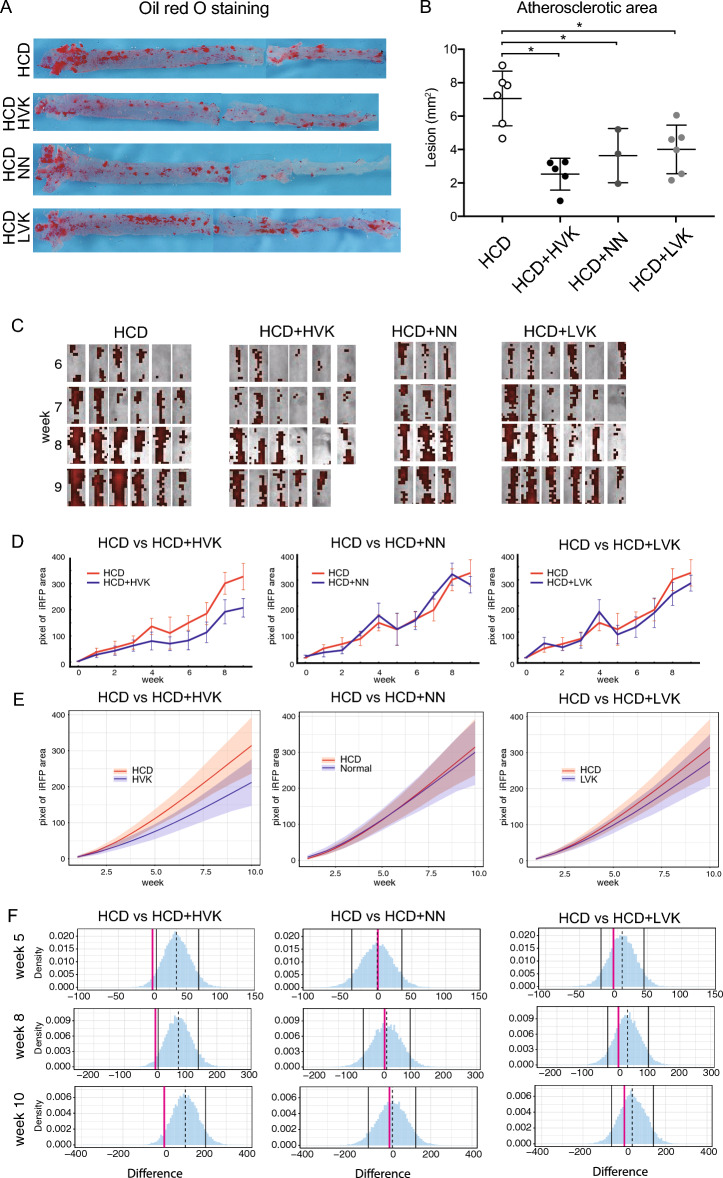
High vitamin K2 natto consumption ameliorates atherosclerotic progression. (**A**) Representative macroscopic image of the atherosclerotic aorta after Oil red O staining. (**B**) Quantified atherosclerotic areas. Means +/− SEM are shown with individual samples (n = 6 for HCD, HCD + HVK, HCD + LVK. n = 3 for HCD + NN). (**C**) Weekly IVIS images of the thoracic area. Each column shows one mouse. (**D**) Line graph of the mean pixel data of iRFP in the chest area for each group over weeks 0–9, comparing HCD with HVK, NN, and LVK. Data are shown as mean + / − SEM. (**E**) Values of iRFP signals of groups in 10 weeks of the induction. Values are calculated by Bayesian statistics with the model described in the Method section. Data are shown as estimated mode (solid lines) with 89% credible interval (bands). (**F**) Comparisons of iRFP signals between groups on week 5, week 8, and week 10. Each graph shows the subtraction from the value of HCD control. Data are shown as estimated mode (dotted line) with 89% credible interval (black lines).

Evaluation of thoracic aortas by Oil red O staining revealed a significant reduction in atherosclerotic lesions in all natto groups (HVK, LVK, NN) compared to the HCD group (*p* < 0.05) (Fig. 1A, B), with a particularly pronounced decrease in the HCD + HVK group.

Furthermore, weekly IVIS imaging of the thoracic region (Fig. 1C, Supplementary Fig. 2C, D) revealed an increase in iRFP fluorescence over time in all groups. Figure 1D illustrates the weekly changes in thoracic iRFP signals, comparing HCD vs HCD + HVK, HCD vs HCD + NN, and HCD vs HCD + LVK. To ascertain whether there are differences in these results, it was determined to analyze them using Bayesian inference. Figure 1E displays the weekly expression intensity of iRFP estimated using Bayesian inference. In the HCD, HCD + NN, and HCD + LVK groups, the expression intensity reached around 100 pixel/area at week 5 and exceeded 300 pixel/area at week 10. However, in the HCD + HVK group, the intensity was approximately 75 pixel/area at week 5 and around 200 pixel/area at week 10. This suggests that the inhibitory effect of HVK on atherosclerosis was observed at an earlier time point than 5 weeks, as indicated by non-invasive observations (Fig. 1E left panel). Additionally, comparison of iRFP signals between groups at 5, 8, and 10 weeks after induction (Fig. 1F) demonstrated significant differences between HCD and HCD + HVK. The graphs represent the difference from the HCD control values, with the estimated mode (dashed line) and 89% confidence interval (black line) displayed.

In parallel, we conducted serum analyses to further elucidate the potential benefits of natto by examining impacts on liver function and lipid profiles. Each group demonstrated an initial increase in liver function markers (AST and ALT) in response to the high-fat diet, but there was no significant subsequent deterioration or improvement in these parameters over the time course. Furthermore, variable K2 content did not appear to significantly influence these liver function indicators (Supplementary Fig. 3A).

As for the lipid profiles, although certain statistical significances were found in serum lipid concentrations, these findings did not establish a consistent pattern (Supplementary Fig. 3B). Our investigation into the activity of lipoprotein lipase (LPL)—an enzyme often associated with suppression of atherosclerosis onset—showed that neither natto nor variable vitamin K2 content had a substantial impact on LPL activity (Supplementary Fig. 3C).

### Effects of natto intake on gut microbiota diversity

**Figure 2:**
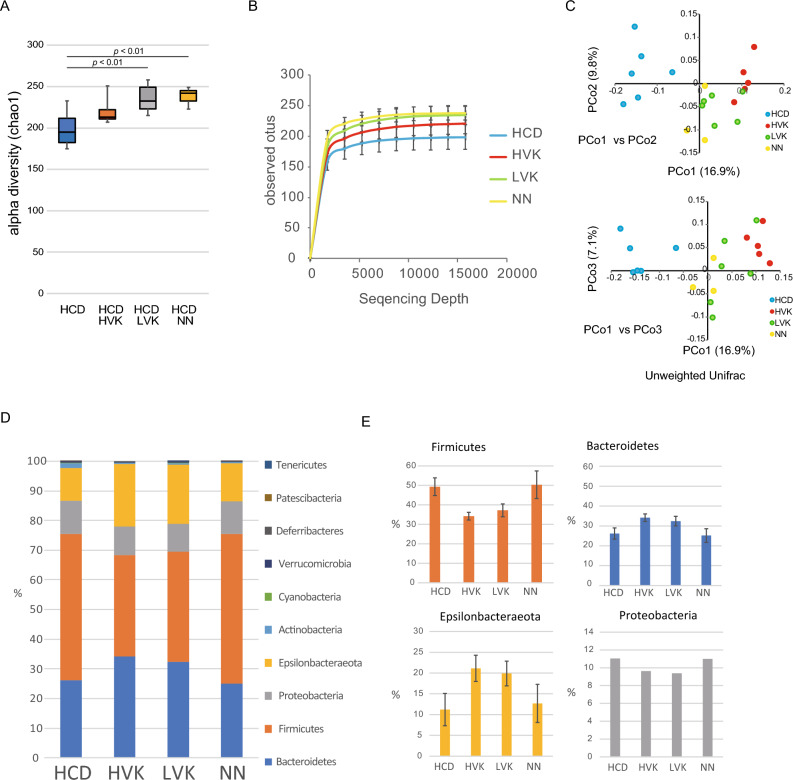
Microbiota diversity was increased after 9 weeks of natto consumption. (**A**) Box plot of alpha diversity (Chao1) of microbiota in each group after 9 weeks of the induction. Data are shown as median with maximum, quartiles (25% and 75%), and minimum. n = 6 for HCD, HCD + HVK, HCD + LVK. n = 3 for HCD + NN. (**B**) Number of observed OTUs with sequencing depth in each group. Graphs are shown as mean +/− SEM. (**C**) Unweighted UniFrac analysis of microbiota in groups 9 weeks after induction. (**D**) The proportion of bacterial families in each group after 9 weeks of the induction. (**E**) The percentages of four major families (Firmicutes, Bacteroidetes, Epsilonbacteraeota, Proteobacteria) in each group. Data are shown as mean +/− SEM. *OTU* operational taxonomic unit. *HCD* High-cholesterol diet. *HVK* High-vitamin K natto. *LVK* Low-vitamin K natto. *NN* Normal natto.

Natto contains *Bacillus subtilis* var. natto, and we studied its effects on the gut microbiota in atherosclerosis-prone mice fed HCD. HCD + HVK, HCD + NN, and HCD + LVK natto groups were compared to the HCD group by cecal collection at 5 and 9 weeks. These contents were subjected to an alpha diversity (chao1) analysis and a rarefaction analysis to evaluate microbiota diversity (Fig. 2A, B). As a result, compared to the HCD group, the number of microbial species significantly increased by roughly 20% in the HCD + NN and HCD + LVK groups, while no significant increase was observed in the HCD + HVK group (Fig. 2A, B). We next conducted a UniFrac analysis to evaluate similarities in gut microbiotas between the natto intake and HCD groups (Fig. 2C). A two-dimensional scatter plot created by the principal coordinate analysis revealed significant differences between the natto intake (red, green, and yellow) and control groups (blue) (Fig. 2C). In addition, when we observed the specific distribution of microbial species in the gut microbiotas, we found differences in microbial composition, particularly in a trend toward decreased Firmicutes in the HCD + HVK and HCD + LVK groups, although it was not significant. A trend towards increased Epsilonbacteraeota was also observed, but it was also not significant (Fig. 2D, E).

### Effects of natto intake on gut microbiota composition

**Figure 3:**
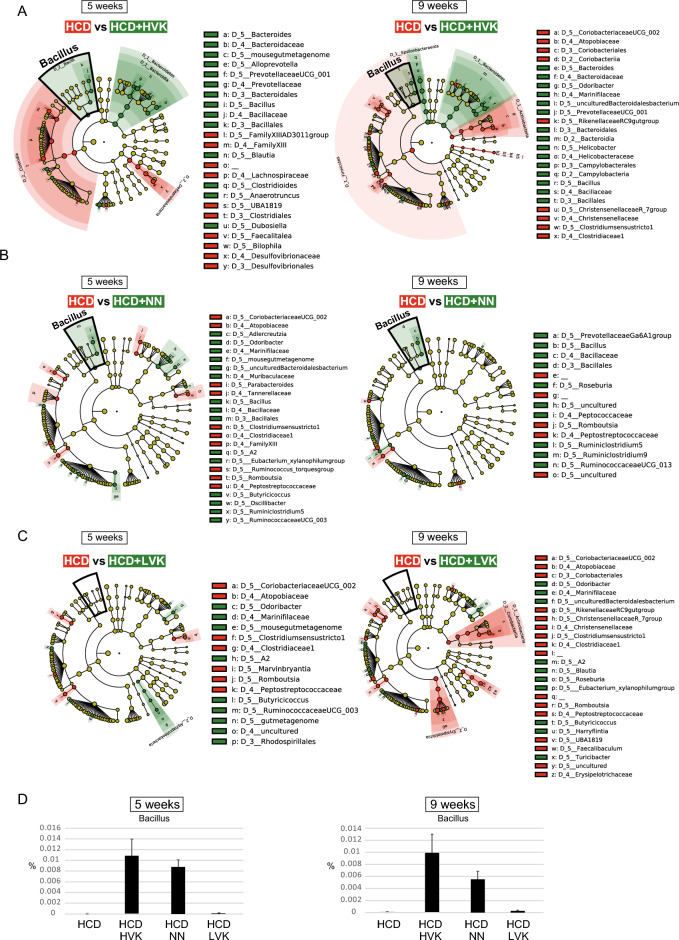
Bacteria of the *Bacillus* genus were enriched after 9 weeks of natto consumption. (**A**–**C**) The cladogram shows the microbial species with significant differences in the groups. Nodes indicate different groups, with the species classification at the level of phylum, class, order, family, and genus shown from the inside to the outside. The red (HCD) and green (HVK in **A**, NN in **B**, LVK in **C**) nodes in the phylogenetic tree represent microbial species enriched in a group, respectively. Yellow nodes represent species without significant differences. Diagrams were made by the LefSe galaxy server. (**D**) The percentage of bacillus genus in each group after 5 weeks (left panel) and 9 weeks (right panel) of induction. Data are shown as mean +/− SEM. *HCD* High-cholesterol diet. *HVK* High-vitamin K natto. *LVK* Low-vitamin K natto. *NN* Normal natto. n = 6 for HCD, HCD + HVK, HCD + LVK. n = 3 for HCD + NN.

To investigate changes in microbial community composition, we performed a group-wise comparative analysis of gut microbiota composition at weeks 5 and 9 post-atherosclerosis induction using color-coded LEfSe. In the HVK group, a high abundance of *Bacillus* spp., including the natto bacterium, was detected at both 5 and 9 weeks, with a decrease in *Clostridium* spp. (Fig. 3A, Supplementary Tables 1, 2). Similarly, in the NN group, a high abundance of *Bacillus* spp. was detected, with little change in other strains (Fig. 3B, Supplementary Tables 3, 4). However, an increase in *Bacillus* spp. could not be confirmed in the LVK group (Fig. 3C, Supplementary Tables 5, 6). These results suggest that the intake of natto alters the composition of gut bacteria, especially in the HVK and NN groups where the proportion of *Bacillus* spp., which was not detected at all in the HCD group, was found to increase by about 0.01% (Fig. 3D).

### Effects of natto consumption on inflammatory markers and cytokine expression

**Figure 4:**
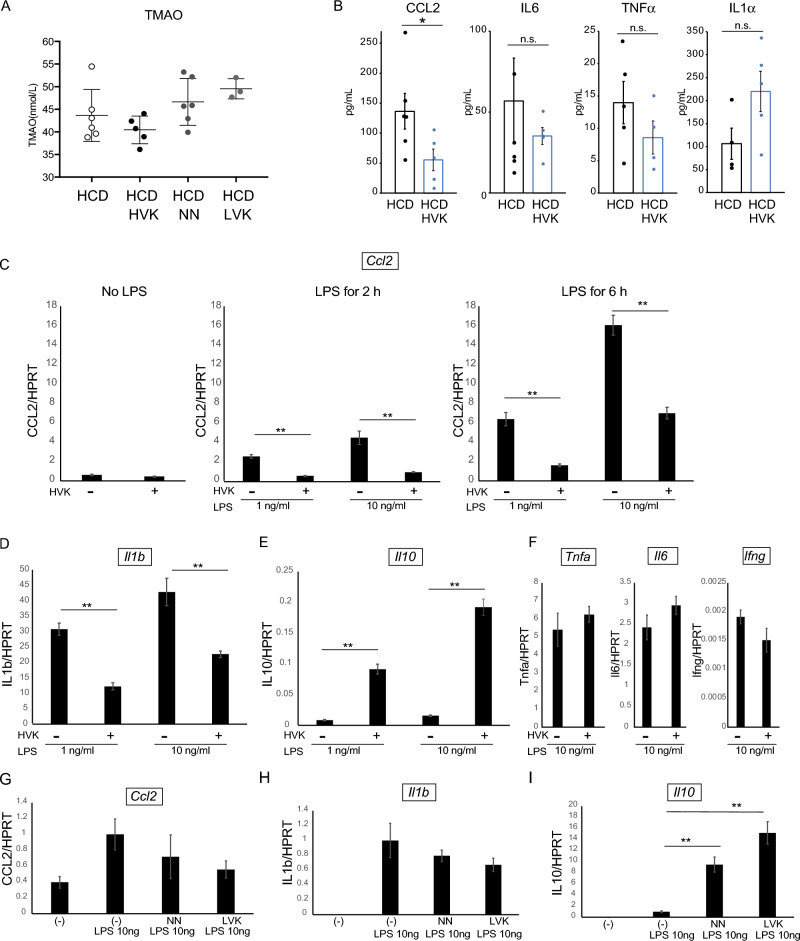
Natto consumption suppresses CCL2 expression in macrophages. (**A**) TMAO concentration in the serum from the mice in each group 9 weeks after induction. n = 6 for HCD, HCD + HVK, HCD + LVK. n = 3 for HCD + NN. (**B**) The concentrations of IL6, CCL2, TNFa in the serum from the mice in HCD and HCD + HVK 9 weeks after induction. n = 6 for HCD, HCD + HVK. (**C**) Relative mRNA expression level of CCL2 in the peritoneal macrophages with or without 3% HVK extract treatment. Values are normalized to the CT values of HPRT. (**D**–**F**) Relative mRNA expression level of IL-1b, IL-10, TNFα, IL6, IFNγ with or without HVK extract supplementation. (**G**–**I**) Relative mRNA expression level of CCL2, IL-1b, IL-10 after NN or LVK extract supplementation. (**C**–**I**) *n* = 6 in each group. Data are shown as mean +/− SEM. HCD: High-cholesterol diet. *HVK* High-vitamin K natto. *LVK* Low-vitamin K natto. *NN* Normal natto.

The observed change in gut microbiota following natto consumption led us to investigate how this alteration might impact atherosclerotic lesions. Trimethylamine N-oxide (TMAO), a metabolite produced by intestinal bacteria via the metabolism of dietary choline and L-carnitine, has been clinically linked to an increased risk of cardiovascular events when present at elevated levels in the bloodstream. To explore this, we measured serum TMAO levels by enzyme-linked immunosorbent assay (ELISA) after 9 weeks of high-cholesterol diet (HCD), supplemented with natto consumption, but found no significant differences from controls (Fig. 4A). With no changes observed in TMAO levels, we sought other potential contributors to atherosclerosis development and thus examined cytokine levels in the serum. Cytokines serve as inflammatory markers, and CCL2, a crucial chemotactic factor for monocytes and macrophages involved in atherosclerotic lesion development, was approximately 60% lower in the HCD + HVK group compared to the HCD group (*p* < 0.05) (Fig. 4B middle panel). However, there were no statistically significant differences observed in the levels of IL-6 and TNFα (Fig. 4B). These findings suggest that the consumption of natto contributes to the suppression of atherosclerotic progression at least partially through the reduction of serum CCL2 levels.

To further investigate the relationship between natto and CCL2 production, we focused on macrophages, which are known to express high levels of CCL2. Peritoneal macrophages collected from sacrificed wild-type mice were treated with 3% HVK natto extract. Subsequently, inflammation was induced by administering LPS at concentrations of 1 ng/ml and 10 ng/ml before comparing the expression levels of *Ccl2* at 2 h and 6 h after administration using RT-qPCR. The results demonstrated that the expression levels of *Ccl2* were significantly reduced at both the 2-h and 6-h time points when 1 ng/ml or 10 ng/ml of LPS was administered (*p* < 0.001) (Fig. 4C). Furthermore, when using 3% LVK and 3% NN natto extracts, although the effects were not as pronounced as with 3% HVK natto extract, both extracts still led to a decrease in *Ccl2* expression (*p* < 0.05) (Fig. 4G).

We also explored the effects of 3% HVK natto extract on the expression levels of other inflammatory cytokines, including IL-1β, IL-10, IL-6, IFNγ, and TNFα. The results indicated that the administration of 3% HVK natto extract significantly decreased the expression levels of *Il1b* (Fig. 4D) and significantly increased the expression levels of *Il10* (Fig. 4E). However, no significant differences were observed in the expression levels of *Il6*, *Ifng*, and *Tnfa* (Fig. 4F). Similarly, when examining 3% LVK and 3% NN extract we observed a significant increase in *Il10* expression (Fig. 4I) but did not observe a significant decrease in *Il1b* expression (Fig. 4H).

Furthermore, when cultured wild-type peritoneal macrophages were exposed to surfactin, the biosurfactant of natto bacteria, *Il10* expression induced by LPS was decreased by approximately 30%. Although no statistically significant differences were observed, both *Ccl2* and *Il1b* showed a decreasing trend (Supplementary Fig. 4A). Additionally, the addition of vitamin K2 in a similar experiment did not lead to significant changes in the expression levels of *Ccl2*, *Il1b*, and *Il10* (Supplementary Fig. 4B). Based on these results, it is evident that the effectiveness of HVK natto cannot be solely explained by the high concentration of vitamin K2. Taken together, our findings indicate that the consumption of HVK natto and its components can modulate the inflammatory cytokine profile.

## Discussion

It is well established that natto intake is inversely correlated with cardiovascular disease mortality in Japanese adults^14^. In addition, nattokinase, the primary active enzyme in natto, has been shown to inhibit the progression of atherosclerosis^15^. Thus, it is unequivocal that natto has anti-atherosclerotic effects. However, there have been no reports investigating the comprehensive effects of natto, including changes in the gut microbiota, impacts at the cellular level, and subsequent effects on the host. In this study, we utilized different types of natto to observe detailed effects on atherosclerotic pathogenesis via near-infrared imaging technology.

In this study, the effects of natto product consumption on atherosclerosis were evaluated using iRFP fluorescent labeling and Oil red O staining. Particularly in the HVK group, a significant inhibitory effect on atherosclerosis was observed through Oil red O staining. Meanwhile, in the NN and LVK groups, this inhibitory effect was weaker but still present. Furthermore, revision using IVIS with iRFP demonstrated that the detection of atherosclerosis inhibition by HVK was feasible, whereas it was challenging in the case of weaker effects, as seen with NN and LVK. This highlights the limitations in the sensitivity and specificity of iRFP, necessitating consideration in future research. Furthermore, our study analyzed the weekly changes in iRFP fluorescence using Bayesian inference. This approach allowed us to obtain more precise information regarding the inhibitory effects on atherosclerosis. Bayesian inference, considering the uncertainty inherent to such data, enabled a more accurate capture of weekly changes. Notably, in the comparison between the HCD and HCD + HVK groups, significant differences in iRFP signals were observed at 5, 8, and 10 weeks.

Our investigation revealed that the HVK natto exhibited the highest values in terms of natto kinase activity, PGA, and bacterial count. NN followed suit, with higher values for these components compared to the LVK natto. Compelling evidence exists that vitamin K2, abundant in natto, is necessary to prevent calcium deposition in blood vessel walls and maintain their flexibility^16^. Additionally, surfactin produced by *Bacillus subtilis* var. natto has been shown to help suppress inflammation and prevent the progression of atherosclerosis^17^. Moreover, polyglutamic acid has demonstrated anti-inflammatory effects^18^, Therefore, we hypothesized that the quantity of these components would directly correlate with the suppression of atherosclerosis, thus anticipating an efficacy sequence of HVK > NN > LVK. However, experimental results interestingly showed that HVK natto had the most significant inhibitory effect, while NN and LVK exhibited equivalent effects. This suggests that the differences in the effects may not be solely due to the amount of components but could also be attributed to differences in the impact on the gut microbiota. Upon a closer examination of the gut microbiota data, the alpha diversity and sequencing depth in the HVK group were not significantly higher compared to the NN and LVK groups (Fig. 2A, B). Here, the unweighted UniFrac analysis showed that the gut microbiota profile of the LVK group was closer to the HVK group than to the NN group while analyses of bacterial family proportions showed similar trends for HVK and LVK, with a decrease in Firmicutes and an increase in Bacteroidetes and Epsilonbacteraeota (Fig. 2D, E). Furthermore, as shown in Fig. 3, the amount of natto bacteria, which is an indicator of surviving natto bacteria, was the lowest in the LVK group, suggesting that the influence of dead bacteria in the LVK group might be significant. In conclusion, based on component analysis, an effect sequence of HVK > NN > LVK was expected, but the gut microbiota data resulted in an actual outcome of HVK > NN = LVK. This implies that LVK may have a more favorable impact on the gut microbiota than expected. To elucidate the unique characteristics that LVK may possess, especially beyond its low vitamin K2 content, further detailed research is deemed necessary.

Previous research has demonstrated that a high-fat diet stimulates inflammatory responses through the CCR2/CCL2 signaling pathway^19^. In contrast, a high-cholesterol diet has been shown to induce short-term acute self-inflammation in the gut, with long-term consumption promoting systemic vascular disease^20^. Additionally, as previous research has emphasized the involvement of high-cholesterol diets in the induction of non-alcoholic steatohepatitis (NASH)^21^, excessive dietary cholesterol appears causative for triggering inflammatory responses. Previous reports indicate that such changes in gut microbiota (dysbiosis) lead to the weakening of tight junction connections in the intestinal epithelium, resulting in leakage of lipopolysaccharides from the intestine and subsequent inflammatory responses^22^. Such a diet promotes systemic inflammatory responses and particularly triggers the infiltration of inflammatory macrophages into the gut environment. Studies using mouse models with knocked-out C–C motif receptor 2 (Ccr2) or intestinal epithelial cell-specific Ccl2 knockout have shown that, under a high-fat, diet-induced, chronic inflammatory condition, there is a decrease in the infiltration of inflammatory macrophages into the colon, reduced intestinal permeability, and inactivation of the colonic inflammasome. This suggests that a decrease in CCL2 expression in the gut can inhibit macrophage infiltration and may have anti-inflammatory effects on distant adipose tissue inflammation. The changes in the gut microbiota due to natto consumption, especially HVK natto which strongly suppresses the production of CCL2, may lower such inflammatory responses. Particularly, the bacteria and their metabolites contained in natto components could strengthen the intestinal barrier function and prevent the leakage of inflammatory cytokines, thereby reducing serum CCL2 concentrations. Thus, a reduction in serum CCL2 levels may be related to improvements in the gut microbiota and, consequently, a suppression of inflammation in the gut and systemic responses. To gain a deeper understanding and confirmation of this association, comprehensive studies that simultaneously evaluate changes in the gut microbiota, macrophage dynamics, and CCL2 levels are necessary.

Interestingly, when vitamin K2 (MK-7) was directly added to macrophages, the suppressive effect was not significant. On the other hand, there are reports of adding MK-4 to microglia, brain-resident macrophages^25^, where it was found to significantly reduce the expression of pro-inflammatory cytokines. Given that MK-7 is metabolized into MK-4, the effect might have been weaker in our experiment where MK-7 was directly applied^26^. Therefore, we believe that the vitamin K2 present in HVK may have also contributed to suppressing intestinal inflammation. In addition, we also examined the effects of surfactin. Surfactin is known for its anti-inflammatory properties yet, in our study, its addition to peritoneal macrophages resulted in CCL2 and IL1b decreases alongside a significant reduction in IL-10 expression. This finding aligns with prior research indicating that various surfactin types can lower IL-10 expression in LPS-induced macrophages, underscoring the response variability based on macrophage type and environmental factors. Notably, the addition of natto markedly increased IL-10 expression, potentially counteracting surfactin's effects. In our experiments, we used commercially available surfactin from *Bacillus subtilis*, but results might vary with surfactin extracted directly from natto bacteria. This area warrants further exploration.

Although we must acknowledge limitations in sample size, our results suggest that natto may possess anti-atherosclerotic activities through multiple pathways, including the modulation of inflammatory cytokines produced by macrophages and the anti-inflammatory action of vitamin K. Further gene expression analysis and cytokine secretion measurements have elucidated that natto suppresses inflammatory activation of macrophages via inhibition of the NF-kB signaling pathway and the NLRP3 inflammasome. Future studies should focus on the evaluation of macrophage activation markers in atherosclerotic lesions, identification and isolation of functional components in natto, and detailed elucidation of their mechanisms of action.

## Materials and methods

### Mouse atherosclerosis imaging model

A strain of iRFP-expressing bone marrow transplanted mice (iRFP → LDLR^−/−^) were generated as previously described^6^. Briefly, 1 × 10^7^ iRFP-expressing bone marrow (BM) cells were transplanted to lethally-irradiated (7 Gy) LDLR^−/−^ mice at 10–12 weeks old. Eight weeks after the transplantation, iRFP fluorescence was confirmed by flow cytometric analysis of peripheral blood. We used chimeric mice with chimerism higher than 90% for all experiments.

All mice were maintained under specific pathogen-free conditions in the laboratory animal resource center of the University of Tsukuba. All experiments complied with relevant Japanese and institutional laws and guidelines and were approved by the University of Tsukuba Institutional Animal Use and Care Committee (Authorization #23–043). This experiment complied with ARRIVE guidelines.

### Feeding

As a basal food, an atherogenic high-cholesterol diet with 1.25% cholesterol (Oriental Yeast Co. Ltd, Japan) was used. The diet was specially designed to express no fluorescence^6^. In this study, three different natto powders, HVK, NN and LVK were prepared (Takano Foods Co., Ltd., Japan). Experimental HCDs received 5% (g/g) natto powder; HCD and natto powder were mixed in powder form and freeze-dried to form approximately 10 g pellets. The freeze-dried pellets were given to the iRFP → LDLR^-/-^ mice for eight weeks to induce atherosclerosis. Control mice received only HCD. Each individual mouse was considered an experimental unit.

### In vivo imaging

An in vivo imaging system (IVIS; Perkin Elmer, USA) was used as the imaging device. Live imaging was conducted from day 0 of atherosclerosis induction and monitored every week. During the imaging, mice were anesthetized by inhalation anesthesia (Perkin Elmer, USA) with isoflurane. The ventral surface was shaved and subjected to imaging. All IVIS images were acquired with excitation/emission wavelengths of 710/760 nm and with an exposure time of 1 s.

### Image analysis of the atherosclerotic area

IVIS images were adjusted to the same minimum and maximum range of the color scale by Living Image Software (Perkin Elmer, USA). The values of the negative controls, which did not show any autofluorescence, were selected and set as the minimum (337 counts) and maximum (1020 counts) values. The region of interest (ROI) was manually traced on the atherosclerotic lesions in the thoracic aorta via Living Image Software and separated by Photoshop software (Adobe Systems, USA). The specific signal area was measured by the area measurement function of ImageJ software (National Institutes of Health, USA). Values within these areas of interest were subjected to Bayesian modeling.

### Statistical analysis of the atherosclerotic area

We employed Bayesian statistical techniques to analyze our in vivo imaging findings because of their ability to handle observational noise and various uncertainties^13^. The model fitting was conducted utilizing Hamiltonian Monte Carlo, specifically its adaptive variant known as the No-U-turn Sampler, within the Rstan environment (Version 2.21.2, GitRev: 2e1f913d3ca3) and R (version 3.6.3; 2020-02-29). Convergence was evaluated by examining the trace plots, employing Gelman and Rubin's convergence diagnostics, and estimating the sufficient number of samples. Any iRFP-positive areas in the iRFP → LDLR^−/−^ mice were modeled by a state-space hierarchical model for each feeding group. Defining the unobservable baseline of the signal area as a time-variable *A*_t,f_ where t denotes the week of observation and f represents the food index (with values 1, 2, 3, and 4 corresponding to HCD, HVK, LVK, and Normal Natto), plus considering the trend *m*_t,f_ along with the total time points *T*, the observed state *Y*_t,f_ can be precisely modeled by accounting for the observational error through a log-normal distribution.

1 $$Y_{t,f} \approx log - normal\left[ {\log \left( {A_{t,f} } \right) - \frac{{\left( {\sigma_{1} } \right)^{2} }}{2}, \sigma_{1} } \right]$$

2 $$\left\{ {\begin{array}{*{20}l} {A_{1,f} = \mu_{1,f} , t = 1} \hfill \\ {A_{2,f} - A_{1,f} = A_{1,f} + \mu_{2,f} , t = 2} \hfill \\ {A_{t,f} - A_{t - 1,f} = A_{t - 1,f} - A_{t - 2,f + } \mu_{t,f} , t \ge 3} \hfill \\ \end{array} } \right.$$

3 $$\mu_{t,f} \approx Normal\left( {0,\sigma_{2} } \right)$$

4 $$t = 1 \ldots T$$

5 $$f = \left\{ {1,2,3,4} \right\}$$

Uniform priors were applied to be weakly informative and conservative. σ1 and σ2 were drawn from standard half-normal distribution.

### Tissue sampling

After every IVIS imaging session, approximately 400 uL of peripheral blood was collected by facial vein puncture. Serum samples were separated by centrifugation at 1500 rpm for 15 min at 24 °C. Atherosclerotic mice were sacrificed after eight weeks of IVIS imaging. The mice were carefully perfused with PBS, and the aortas were dissected under a stereoscopic microscope and immediately transferred into 4% PFA at 4 °C. Appendix contents were collected for microbiota analysis.

### Oil red O staining analysis

After the dissection, the aortas were sent to Japan SLC Inc. for Oil red O (ORO) staining. The aortas were stained with ORO, carefully mounted on black paper, and photographed. ORO-positive areas were analyzed by Photoshop and ImageJ software.

### Serum lipoprotein species analysis

The serum obtained as described above was sent to Skylight Biotech Co., Ltd. For lipoprotein analysis without breaking the cold chain. Chylomicron, VLDL, LDL, and HDL proportions were measured.

### Serum cytokine analysis

The serum obtained as described above was sent to Eurofins GeneticLab Co., Ltd. For serum cytokine analysis without breaking the cold chain. IFNg, IL-1a, IL-1b, IL-6, IL-10, CCL2, and TNFα concentrations were analyzed.

### Gut microbiota analysis

Appendix contents obtained as described above were sent to Metagen, Inc. for gut microbiota analysis without breaking the cold chain. The data was analyzed and visualized with Microsoft Excel and Linear discriminant analysis effect size (LEfSe). Data were reformatted in Excel for LEfSe, written to a tab-separated text file, and then uploaded to the LEfSe galaxy server^27^. The default statistical parameters were used in the study to generate LDA scores and the LDA cladogram.

### Macrophage collection and LPS assay

To induce macrophage infiltration, 2 mL of thioglycolate medium (Becton Dickinson, Cat#211716) was intraperitoneally injected. Three days post-injection, the mice were sacrificed, and the peritoneal cavity was flushed with 10 mL of PBS to collect cells, including macrophages.

The cells were collected by centrifugation (1000 rpm, 10 min, 4 °C) and resuspended in RPMI (Sigma Aldrich, Cat#R8758) supplemented with 10%FBS (Gibco, Cat#10270106) medium. After the cell count, 1 × 10^6^ cells per well in 2 mL medium were seeded and incubated at 37 °C, 5% CO_2_ for an hour. After the incubation, the medium, including floating cells, was removed, and macrophages were subjected to an LPS assay.

Natto extracts for immunostimulation were prepared as follows: 1 g of dried natto was suspended in 9 g of distilled water and centrifuged at 3000 × *g* for 10 min. Supernatant was collected and diluted with distilled water 10 times before passing through a 0.22 μm filter. After 24 h of recovery, natto extract was added as 3% of medium for 24 h. Then, lipopolysaccharide (Sigma Aldrich) was added at 1 ng/mL or 10 ng/mL to stimulate macrophages. After two and six hours of stimulation, cells were lysed with Isogen (Nippon Gene, Cat#315–02,504) to prepare total RNA for the RT-qPCR analysis.

### RT-qPCR

Total RNA was collected with an Isogen Kit (Nippon Gene, Tokyo, Japan, Cat. No. 311-02501). The cDNA was synthesized with a QuantiTect Reverse Transcription Kit (Qiagen, Hilden, Germany, Cat. No. 205313). Gene expression levels were determined using RT-PCR performed on a Thermal Cycler Dice Real Time System Single TP850 (Takara Bio Inc., Shiga, Japan) with THUNDERBIRD^®^ SYBR^®^ qPCR Mix (TOYOBO Co., Ltd., Osaka, Japan, Cat.No. QPS-201). The mRNA levels were normalized to that of Hprt. The primer sequences are as follows:

Hprt: F: 5′-CAAACTTTGCTTTCCCTGGT-3′, R: 5′-CAAGGGCATATCCAACAACA-3′.

Mcp-1: F: 5′-TGTTGGCTCAGCCAGATGCA-3′, R: 5′-AGCCTACTCATTGGGATCATCTTG-3′.

IL1-b: F: 5′-GAGCTGAAAGCTCTCCACCTCA-3′, R: 5′-TCGTTGCTTGGCTCCTTGTAC-3′.

IL-10: F: 5′-CCAGGGAGATCCTTTGATGA-3′, R: 5′-CATTCCCAGAGGAATTGCAT-3′.

### Statistics

Graphs are presented as mean ± SEM, as indicated in figure legends. Significance was calculated using GraphPad Prism6 software by either Student’s t-test or one-way ANOVA with Tukey’s test: *p < 0.05, **p < 0.01, ***p < 0.001, ns, not significant.

### Supplementary Information

Supplementary Figures.

Supplementary Tables.

## Data Availability

All data generated or analyzed during this study are included in this published article (and its Supplementary Information files).

## Code availability

The code used for data analysis is publicly available and can be accessed at https://github.com/hamamichhi/Natto-iRFP-signals.
