# Supplementary material for: Natto consumption suppresses atherosclerotic plaque progression in LDL receptor-deficient mice transplanted with iRFP-expressing hematopoietic cells
Source: Sci Rep. 2023 Dec 18;13:22469. doi: 10.1038/s41598-023-48562-y (PMC10728071; doi:10.1038/s41598-023-48562-y)
Supplement: Supplementary file 1 — Supplementary Figures. [file 41598_2023_48562_MOESM1_ESM.pdf]

Supplementary Fig. 1

A

	HCD	HCD+HVK	HCD+NN	HCD+LVK
Water	8.10%	8.20%	8.20%	8.10%
Protein	17.10%	18.30%	18.30%	18.30%
Fat	16.10%	16.30%	16.30%	16.40%
Fiber	<0.1%	0.40%	0.40%	0.20%
Ash	3.40%	3.50%	3.50%	3.40%
Soluble non-nitrogenous substances	55.30%	53.30%	53.30%	53.60%
Menaquinone-7 (Vitamin K2)	Not detected	199 μg /100 g	93 μg /100 g	30 μg /100 g

B

		HVK natto	Normal natto	LVK natto
Natto Kinase activity	(FU/g)	150	85	32
PGA	(mg/g)	13.4	11.5	6.8
Bacterium count	(10^6 cfu/g)	19500	13700	638
VK2 in natto water extract	(ng/mL)	39.5	12.3	4.9

Supplementary Fig. 1: Characterization of each diet.

(A) Nutritional assessment of each diet.

(B) Values of Natto kinase activity, PGA, Bacterium count, Vitamin K2 of each natto types.

Supplementary Fig. 2

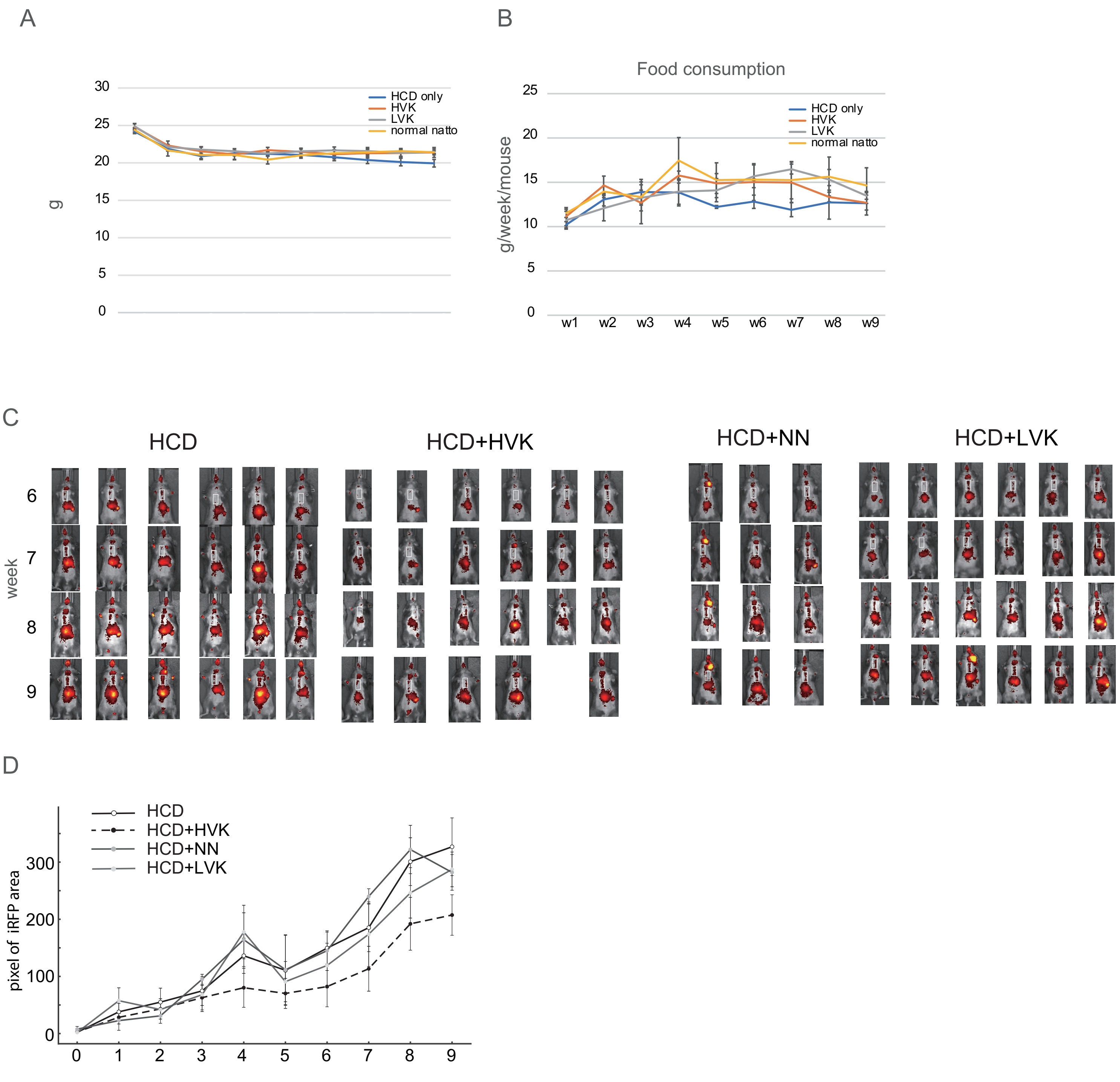

Supplementary Fig. 2: Gross analysis after the diet initiation.
(A) Body weight changes in each group post diet initiation.
(B) Food consumption patterns of each group post diet initiation.
(C) In vivo imaging (IVIS) of each group's mice.
(D) Temporal changes in the mean iRFP signal in the chest area of the IVIS data for each group
Data are shown as mean +/- SEM.

Supplementary Fig. 3

A

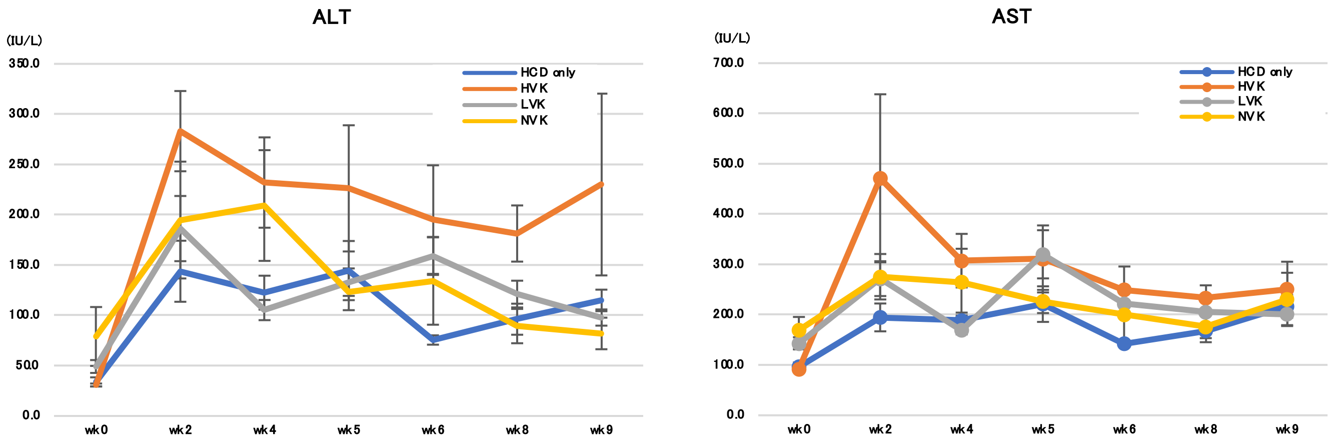

B

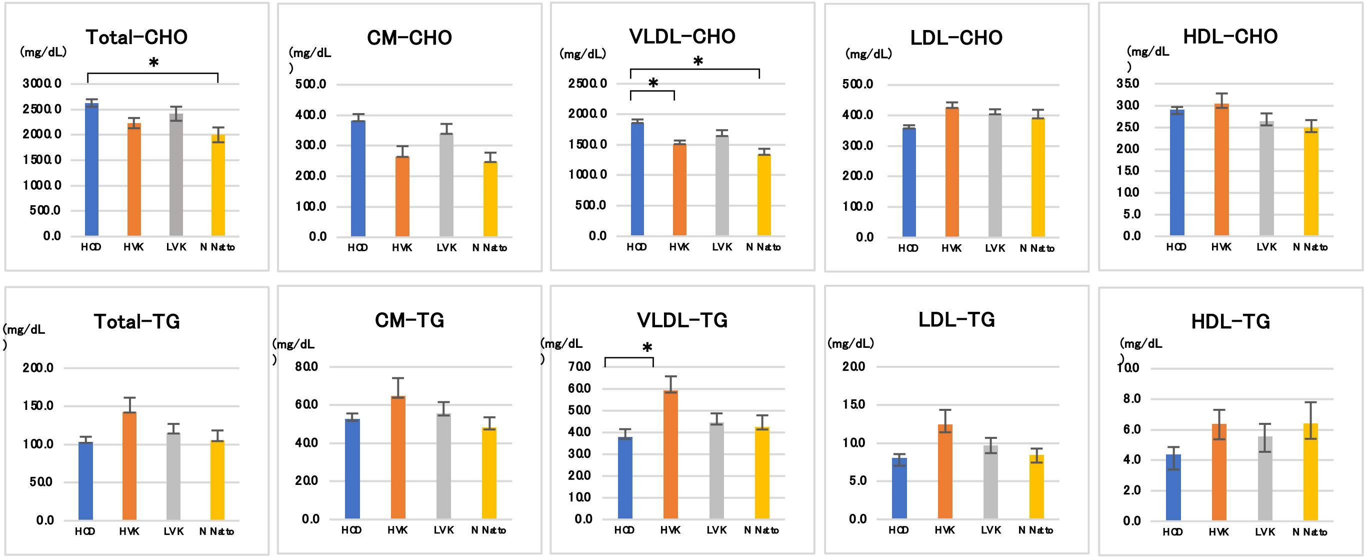

C

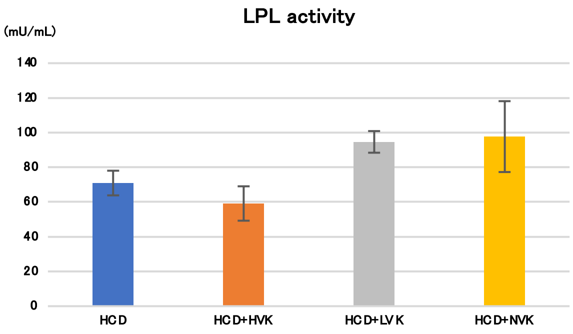

Supplementary Fig. 3: Serum parameters after the diet initiation.
(A) Serum levels of liver function markers (AST and ALT) in each group. (B) Lipid profile analysis of serum samples from each group. (C) Assessment of lipoprotein lipase (LPL) activity in serum samples from each group.The depicted data are representative of at least two independent experiments, and are presented as means ± s.e.m. *, $p < 0.05$ (ANOVA).

Supplementary Fig. 4

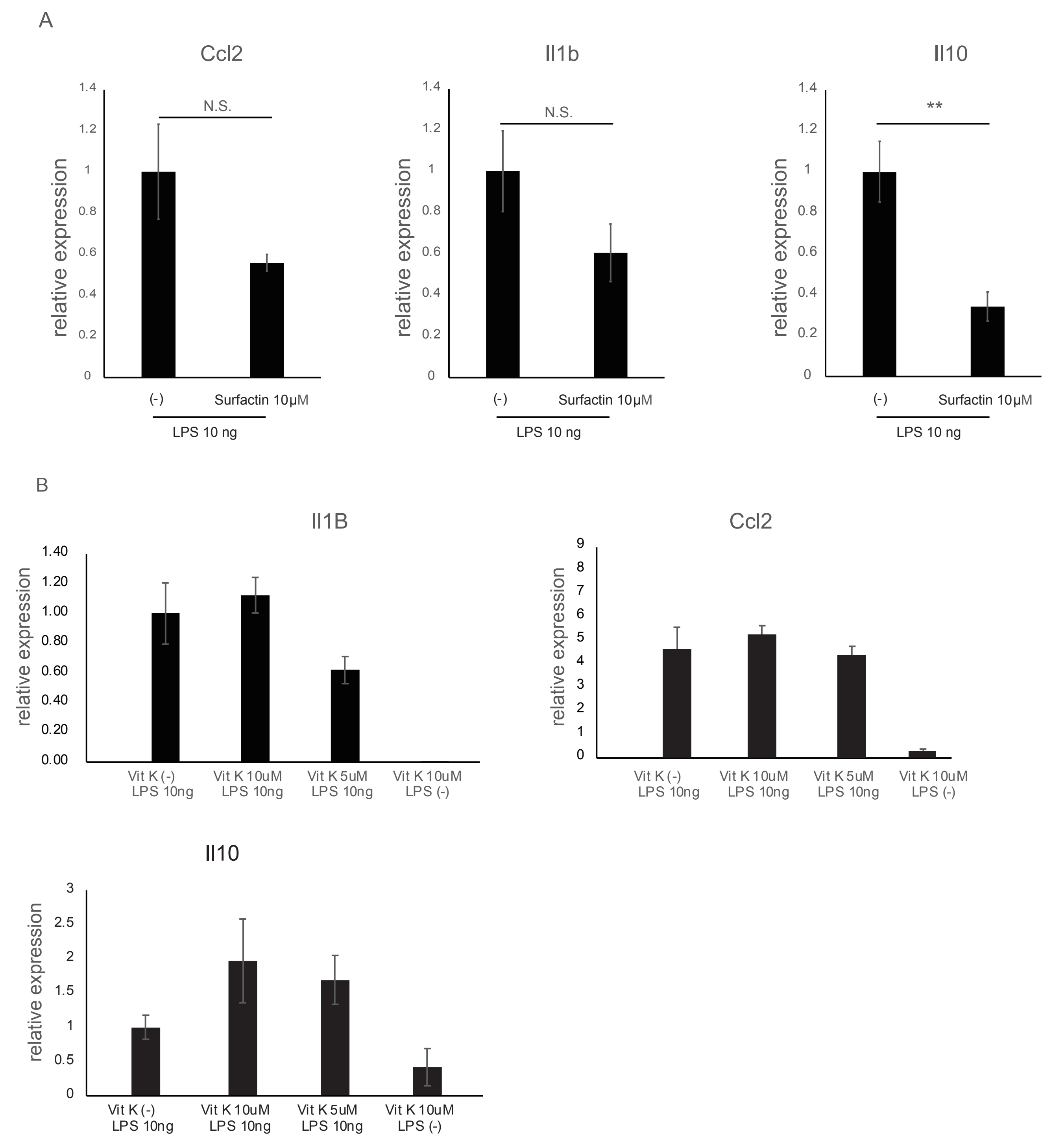

Supplementary Fig. 4: mRNA expression of inflammatory genes.

(A) Expression analysis of Ccl2, Il1b, and Il10 in peritoneal macrophages treated with LPS and with or without surfactin, as determined by qRT-PCR.

(B) Expression analysis of Il1b, Ccl2, and Il10 in peritoneal macrophages treated with LPS and MK-7 (Vitamin K2), as determined by qRT-PCR. The depicted data are representative of at least two independent experiments, and are presented as means ± s.e.m. No significant differences were observed (A: Welch' s t-test, B: ANOVA).
